# An Exploratory Study of ABT-806, a Tumor-Specific Anti-EGFR Antibody, in Subjects with Recurrent or Metastatic Head and Neck and Other Squamous Cell Carcinomas

**DOI:** 10.3390/cancers18203313

**Published:** 2026-10-14

**Authors:** Hui K. Gan, Sagun Parakh, Malaka Ameratunga, Anis Hamid, George Iatropoulos, Michelle Beecher, Diana Cao, Kerryn Westcott, Jaren Caine, Jodie Palmer, Edward B. Reilly, Fiona E. Scott, Andrew M. Scott

**Affiliations:** 1 Medical Oncology, Austin Hospital, 145 Studley Road, Heidelberg, Melbourne, VIC 3084, Australia; sagun.parakh@onjcri.org.au (S.P.); malaka.ameratunga@monash.edu (M.A.); hamida1@mskcc.org (A.H.); georgios.iatropoulos@monashhealth.org (G.I.); michelle.beecher@austin.org.au (M.B.); jaren.caine@austin.org.au (J.C.); 2 Tumor Targeting Program, Olivia Newton-John Cancer Research Institute, Melbourne, VIC 3084, Australia; diana.cao@onjcri.org.au (D.C.); kerryn.westcott@onjcri.org.au (K.W.); jodie.palmer@onjcri.org.au (J.P.); fiona.scott@onjcri.org.au (F.E.S.); andrew.scott@onjcri.org.au (A.M.S.); 3 School of Cancer Medicine, La Trobe University, Melbourne, VIC 3084, Australia; 4 Oncology Discovery, AbbVie Inc., North Chicago, IL 60064, USA; ed.reilly@abbvie.com; 5 Department of Molecular Imaging and Therapy, Austin Health, Melbourne, VIC 3084, Australia; 6 Faculty of Medicine, Melbourne University, Melbourne, VIC 3010, Australia

**Keywords:** EGFR, ABT-806, squamous cell carcinoma

## Abstract

**Introduction:** Despite their benefit, current anti-EGFR therapies are limited by narrow efficacy profiles due to dose-limiting toxicities. This phase 2 open-label study evaluated the efficacy and safety of ABT-806, an EGFR-targeting antibody that is tumor specific, in patients with advanced EGFR-expressing tumors including head and neck cancers (H&NC) and squamous cell carcinoma (SCC) of other sites. **Methods:** Patients with advanced H&NC (cohort A) or SCC of other sites (cohort B) were enrolled in the study. ABT-806 was administered as monotherapy at a dose of 24 mg/kg intravenously using a twice weekly dosing schedule until unacceptable toxicity and/or evidence of disease progression. Adverse events (AEs) were graded using the National Cancer Institute Common Terminology Criteria for Adverse Events v4.0; tumor response was assessed using the Response Evaluation criteria in Solid Tumors v1.1. EGFR status was assessed with mAb806 and EGFRvIII immunohistochemistry and correlated with outcomes. **Results:** ABT-806 was well tolerated with no grade 3+ treatment-related AEs. Disease stabilization was observed in two patients (14%) and for a median duration of 19.5 weeks. This included one patient from cohort A and one patient from cohort B. Progression-free survival and overall survival was not significantly different between the two cohorts. **Conclusions:** Although clinical activity was limited in this small, heavily pre-treated, and biomarker-unselected population, ABT-806 demonstrated an excellent safety profile with minimal classical EGFR-related toxicity. These findings support the development of more tumor-specific EGFR antibodies for use, particularly in combinatorial regimens.

## Simple Summary

The epidermal growth factor receptor (EGFR) is a protein found on the surface of cells that can promote cancer growth. Existing drugs that target EGFR often cause debilitating side effects such as skin rashes and diarrhea, which can limit their use. We have developed a new treatment called ABT-806 that is designed to recognize a form of EGFR found mainly on cancer cells while largely avoiding healthy tissues. In this paper, we show that ABT-806 is very well tolerated and causes very few side effects. The trial was closed early, resulting in a relatively small number of participants. The findings support continued development of more tumor-specific EGFR-targeted therapies and may help guide future research to safer, more precise cancer treatments.

## 1. Introduction

Improvements in the tolerability and efficacy of cancer treatment have come from the use of more selective drugs that specifically target cancer-associated proteins. The EGFR receptor, a member of the ErbB family, represents one such prototypical protein [1]. The ErbB family, in addition to EGFR, includes ErbB2 (Neu, HER2), ErbB3 (HER3), and ErbB4 (HER4), which are homologous transmembrane proteins involved in various downstream signaling pathways affecting cell differentiation and proliferation [2]. EGFR-directed therapies including cetuximab, panitumumab, and necitumumab have gained widespread use in a broad variety of different cancer types, including lung cancer, head and neck cancer (H&NC), colon cancer, and pancreatic cancer [3,4,5,6]. Squamous cell carcinomas (SCCs) frequently express EGFR and tend to be more sensitive to EGFR inhibition than non-squamous histologies [7]. Despite their benefit, cetuximab, panitumumab, and necitumumab, which all bind to extracellular domain III of EGFR, are limited by narrow efficacy profiles due to dose-limiting toxicities and a lack of reliable biomarkers to predict response. Common toxicities encountered with these therapies include skin rash, diarrhea, stomatitis, fatigue, and electrolyte disturbances [8,9]. Furthermore, resistance is common and may be mediated by multiple mechanisms including mutations in *EGFR*, *KRAS*, *BRAF*, PI3K, and PTEN [10,11].

ABT-806 is a veneered “humanized” recombinant IgG1κ antibody that is specific for a unique epitope of human EGFR. This specific EGFR epitope, identified in a disulfide bonded loop (amino acids 287–302) in EGFR domain II, is available for binding only on tumor cells with specific conditions favoring EGFR over-activity, including the presence of the EGFRde2-7 (also known as EGFRvIII) mutant as well as tumors with amplification of the wild-type receptors [12,13,14]. In contrast, the epitope is largely inaccessible when EGFR is expressed at normal physiological levels, resulting in limited binding to normal tissues [12]. ABT-806 is therefore expected to be a more tumor-specific antibody, resulting in a better therapeutic threshold in the treatment of EGFR-expressing tumors, with limited toxicity as confirmed in phase 1 studies [13]. Furthermore, ABT-806 has shown early signals of activity in SCC of the skin and H&NC [13]. It is therefore postulated that ABT-806 will be a well-tolerated and active agent in patients with high EGFR expression such as H&NC and SCC from other sites.

Described herein is a phase 2 open-label study evaluating the efficacy (DCR, RR, PFS, and OS) and safety of ABT-806 in subjects with advanced (recurrent or metastatic disease) (i) H&NC or (ii) SCC of other sites.

## 2. Methods

### 2.1. Study Objectives

The primary objective of the study was to determine the efficacy of ABT-806 in patients with advanced squamous cell carcinoma originating from the head and neck or other sites of the body. The secondary objectives included (i) determination of the toxicity of ABT-806 in patients with recurrent or metastatic H&NC; (ii) determination of the response rate (RR), progression-free survival (PFS), and overall survival (OS) of these patients; and (iii) exploration of the use of EGFR phenotyping as a biomarker in patients treated with ABT-806-based therapy.

### 2.2. Study Population

Patients with advanced (recurrent or metastatic disease) histologically or cytologically proven (i) H&NC (cohort A) or (ii) SCC of other sites (cohort B) were enrolled in the study. Major inclusion criteria included Eastern Cooperative Oncology Group (ECOG) score ≤2 and adequate organ function. Patients with H&NC must have received prior platinum-based chemotherapy for recurrent/metastatic disease or have progressed within six months of concurrent chemo-radiotherapy incorporating cisplatin-based chemotherapy and retained adequate organ function. Key exclusion criteria included symptomatic or active cerebral metastases and prior EGFR-directed monoclonal antibody (mAb) therapy within a period of 4 weeks prior to the first dose of ABT-806.

### 2.3. Study Design

This was a single site phase 2 open-label investigator-initiated study with a companion biomarker study. ABT-806 was administered at a dose of 24 mg/kg intravenously every two weeks. The dose selected for this study was informed by data from the first-in-human phase 1 study [13]. ABT-806 infusion was preceded approximately 30 min prior with promethazine 12.5–25 mg IV and 500–1000 mg paracetamol. Patients continued on this regimen provided they tolerated the drug, showed no evidence of disease progression, and did not meet any of the criteria for subject discontinuation. The date of first patient enrolment is 10 February 2014, and the date of last patient follow-up was 16 October 2016. Recruitment to this study was halted early after an interim review of the efficacy data and funding reasons.

### 2.4. Safety and Response Assessments

Adverse events (AEs) were graded as defined by the National Cancer Institute (NCI) Common Terminology Criteria for Adverse Events (CTCAE) version 4.0 [15]. Safety was assessed through the recording of AEs; laboratory tests (hematology, serum chemistry, and urinalysis); and physical examinations including assessment of injection site and assessment of vital signs. Tumor measurements were obtained with computed tomography (CT) scan at screening and every 8 weeks thereafter, and tumor responses were determined in accordance with RECIST version 1.1 [16]. Survival information was collected monthly after the last study visit for a period of up to 1 year from when the subject is discontinued from the study.

### 2.5. Immunohistochemistry

EGFRvIII expression and ABT-806 binding were evaluated using immunohistochemistry (IHC). Slides were deparaffinized and rehydrated. Antigen retrieval was achieved, and endogenous peroxidase and nonspecific protein-binding sites were blocked using SuperBlocking Buffer (#37515, Thermofisher, Waltham, MA, USA). Samples were subsequently incubated in the primary antibody anti-EGFRvIII (D6T2Q, #64952, Cell Signalling, Danvers, MA, USA) at 1.12 µg/mL and monoclonal antibody mAb806 (mAb806; LICR BDF; in-house) at 1 µg/mL. The murine homolog of ABT-806, mAb806 has previously been extensively used in our laboratory for immunohistochemistry [17]. Novolink poly-HRP secondary antibody was used for mAb806, and Dako Envision anti-rabbit-HRP (#K4003, Carpinteria, CA, USA) was used for EGFRvIII detection with DAB as chromogen (Dako, Carpinteria, CA, USA) and hematoxylin counterstaining. IHC slides were scanned using the Aperio ScanScope XT (Leica Biosystems, San Diego, CA, USA) and analyzed using Aperio ImageScope software 12.3.3 and the Aperio IHC Nuclear and Cytoplasmic Algorithm, which scores IHC expression as negative (0), weak (1+), moderate (2+), or strong. (3+) [18]. All IHC scoring was performed blinded to clinical outcome.

### 2.6. Statistical Analysis

Using a Fleming’s one-stage design with alpha of 0.05, beta of 0.2, p0 = 0.20, and p1 = 0.45, a sample size of 19 patients was recommended for cohort 1 based on an 8-week disease control rate (DCR) in patients with H&NC. A pragmatic sample size of 20 patients was utilized for cohort B. This would be equivalent to a Fleming one-stage design with alpha of 0.05, beta of 0.2, p0 = 0.10, and p1 = 0.30 using an 8-week DCR in patients with non-H&NC with squamous cell carcinoma. A whole spectrum of toxicity was collected and assessed with regard to safety.

All statistical analyses were performed using Graphpad Prism 8.4.3. Descriptive statistics are provided for demographic variables. PFS and OS were analyzed with Kaplan–Meier methodology using data from all dosed subjects. Participants who received one dose or more were included in the analysis.

This study was approved by the Institutional Ethics Committee (IEC) at Austin Health (Approval number: HREC/13/Austin/253). The study was conducted in accordance with the principles of the Declaration of Helsinki (fourth revision, 1996) and the International Conference on Harmonisation (ICH) Good Clinical Practice (GCP) Guidelines. Written informed consent was obtained from all patients, and all methods were performed in accordance with the protocol-specified guidelines and regulations.

Early closure of the study resulted in enrolment of only 14 patients in the study, with 12 patients receiving treatment. No formal protocol-specified interim efficacy analysis or stopping boundary was incorporated into the study design.

## 3. Results

### 3.1. Demographics

A total of 16 patients were screened and 14 patients were enrolled in the study. Two patients withdrew prior to cycle 1 (1 non-compliance, 1 rapid progressive disease) and were excluded from further analysis. Seven patients had H&NC (cohort A), and five patients had SCC of other sites (cohort B). All 12 patients completed at least one cycle of treatment. Baseline patient characteristics are summarized in Table 1. The median age of the patients was 75.0 years (range 62–85). The majority were male (75%), and most (67%) had ≥2 prior lines of treatment. In patients enrolled in cohort B, the primary cancer diagnosis was lung, esophageal, anal, and cutaneous SCC. Individual patient disease characteristics is provided in Supplementary Table S1.

### 3.2. Safety

Eleven of the 12 patients experienced treatment-emergent AEs; treatment-related AEs included an infusion reaction (*n* = 1, grade 2), chills (*n* = 1, grade 1) and rash (*n* = 1, grade 1). Other AEs considered possibly related to drug included grade 1 findings of fatigue (*n* = 3), diarrhea (*n* = 1), and hypomagnesemia (*n* = 1). No patient required a dose reduction or dose delay, and there were no treatment-related deaths. Significantly, skin rash commonly seen with other anti-EGFR therapies was rarely observed with ABT-806. A table of all the toxicities are provided in Supplementary Table S2.

### 3.3. Efficacy

**Figure 1:**
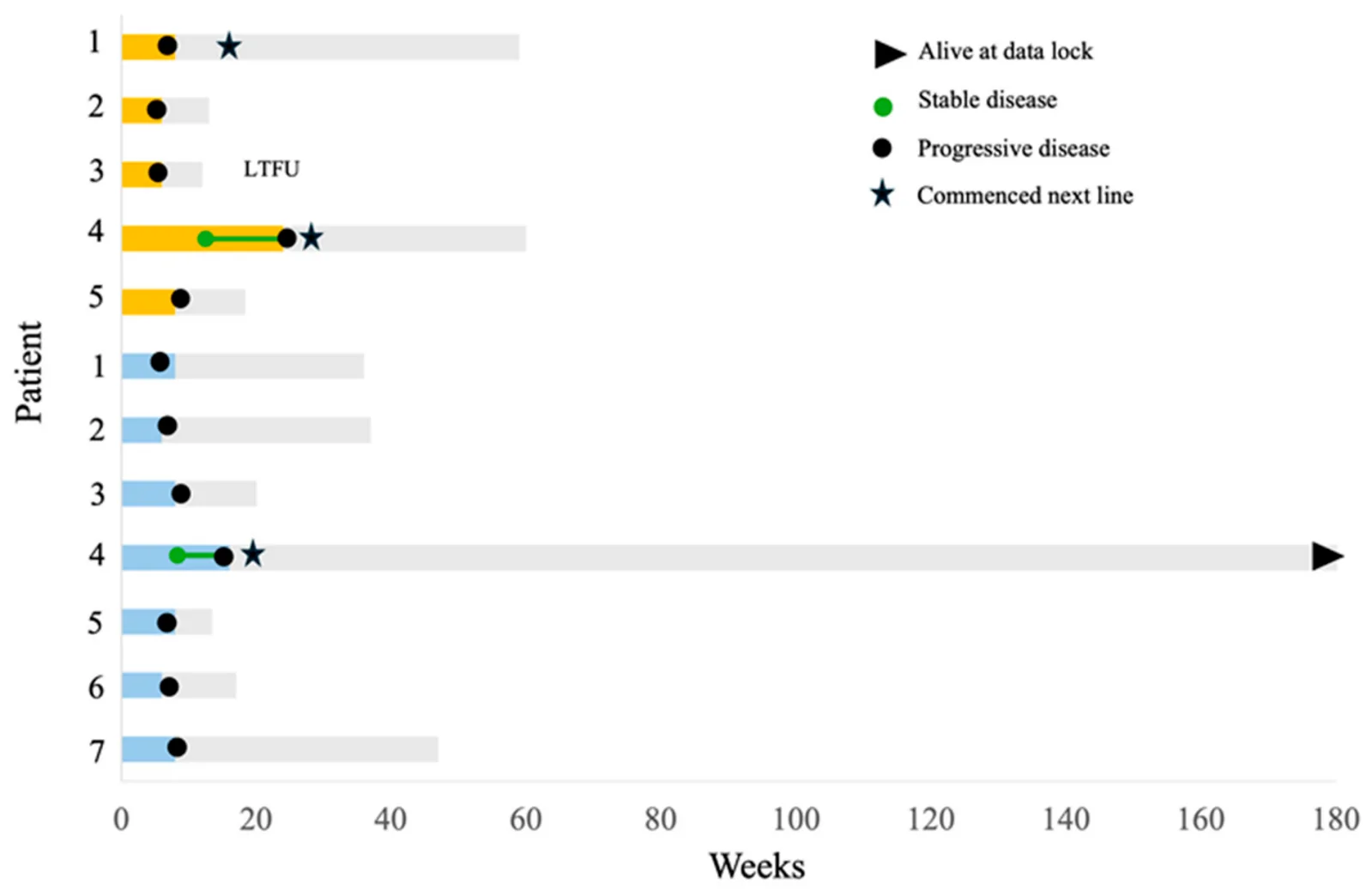
A swimmer plot of patients across the two cohorts illustrates duration of treatment (colored bar), survival follow-up (grey bar), and radiological assessment of disease response (SD or PD). Cohort A (blue bar) and cohort B (yellow bar).

**Figure 2:**
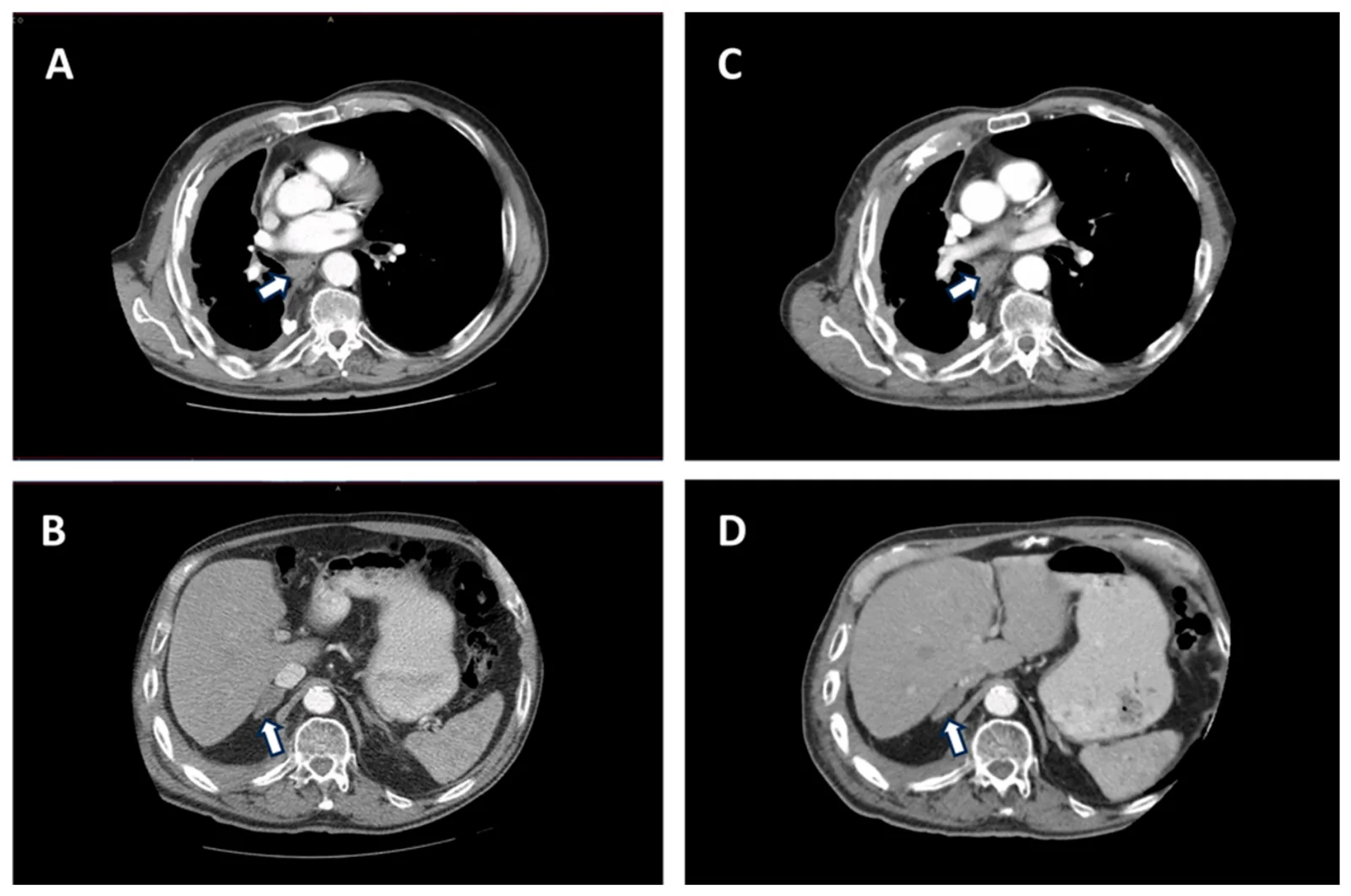
(**A**) Subcarinal node and (**B**) right adrenal mass (arrows) at study enrolment and restaging CT showing (**C**) a stable subcarinal node and (**D**) right adrenal mass (cohort A, patient #4).

The overall DCR was 16.7% (2/12 patients; 95% CI 2.1–48.4%), comprising one patient with stable disease in each cohort. The DCR was 14.3% (1/7 patients; 95% CI 0.4% to 57.9%) in cohort A and 20.0% (1/5 patients; CI 0.5% to 71.6%) in cohort B. The best responses in this study were stable disease observed in two patients, one each from cohorts A and B, both observed at the first restaging scan (Figure 1). The median duration of disease stabilization for these two participants was 19.5 weeks. The patient in cohort A (patient #4) was a patient with metastatic parotid gland squamous cell carcinoma (p16 negative), who developed metastatic recurrence in the lungs following post-operative radiotherapy. The patient received a total of eight cycles of treatment before stopping treatment due to cancer progression in the bones (Figure 2). The patient in cohort B (patient #4) had metastatic non-small cell lung cancer (squamous cell carcinoma; PD-L1 negative), with metastases to right pleura, lymph nodes, and bone. The patient had platinum-doublet chemotherapy prior to enrolling in the study. The patient completed 10 cycles of ABT-806 treatment before stopping treatment due to cancer progression.

The median PFS values in cohorts A and B were 53 days (95% CI: 42.0–117.0) and 42 days (95% CI: 28–166), respectively, with no statistical difference observed between the two cohorts (*p* = 0.88). The median overall survival values in cohorts A and B were 254 days (95% CI 80–1433) and 401 days (95% CI 57–752), respectively, with no significant differences between cohorts (*p* = 0.884).

### 3.4. Biomarker

**Figure 3:**
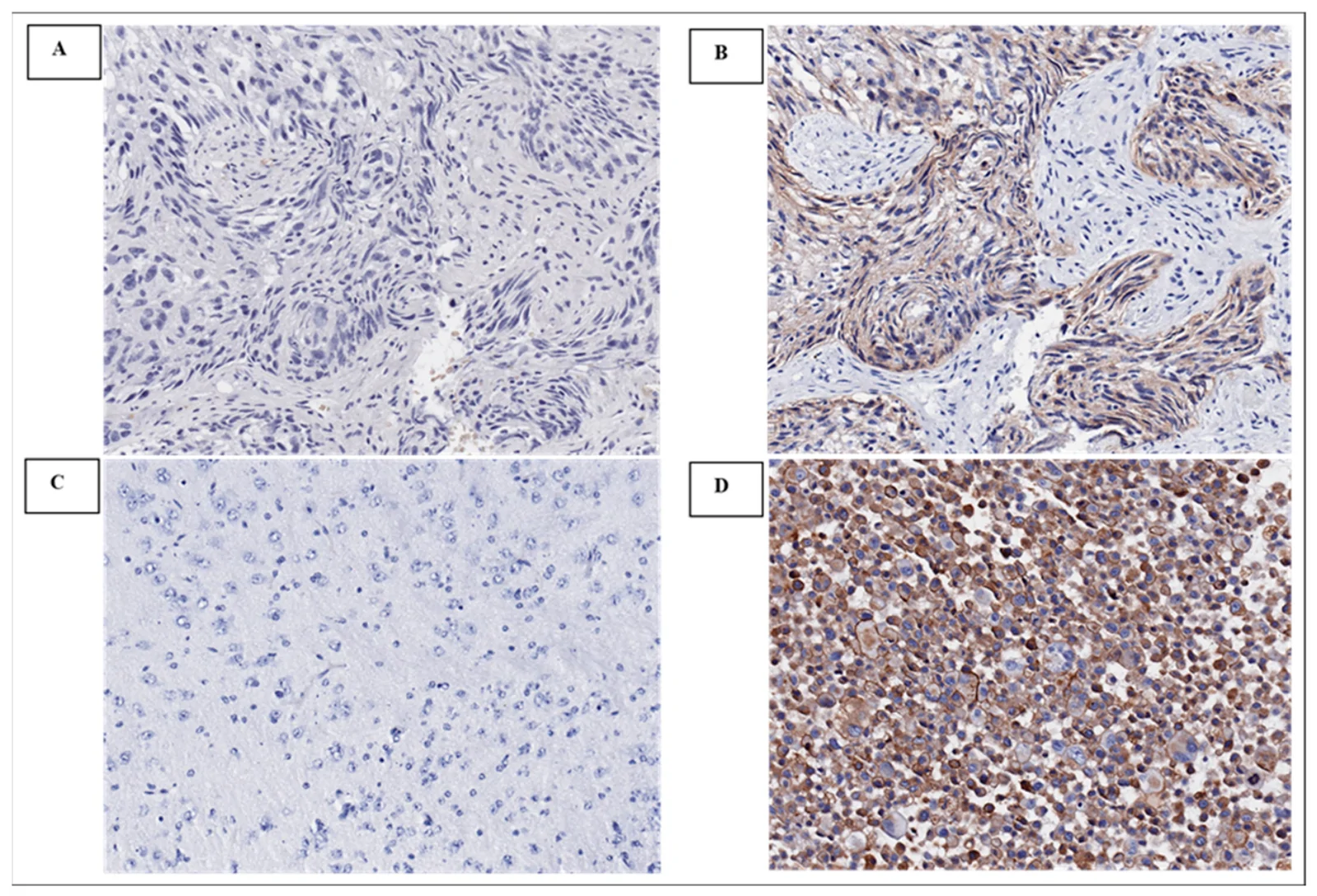
EGFRvIII and mAb806 expression using immunohistochemistry in patient (cohort A, #4) with disease stabilization with ABT-806 treatment (×20 magnification): (**A**) EGFRvIII, (**B**) mAb806 epitope immunoreactivity, (**C**) mAb806-negative control, (**D**) mAb806-positive control.

Of the 12 treated patients, seven had archival tissue for biomarker evaluation. Conformational and mutation forms of EGFR were detected with IHC using mAb806. Four patients had mAb806 staining: one with 1+ staining, two with 2+ staining, and one patient with 3+ staining (Table 2). In general, there was no clear association between 806 protein expression and response or duration of treatment. However, the one patient in cohort A (patient #4) who had disease stabilization with mAb806 treatment had 3+ staining for mAb806 expression (Figure 3). No staining for EGFRvIII was observed with the exception of one patient with 2+ staining (cohort B, patient #1; Table 2).

## 4. Discussion

ABT-806 was well-tolerated and did not exhibit the toxicities commonly associated with other anti-EGFR antibodies. Cetuximab, a chimeric IgG1 mAb, and panitumumab, a fully human IgG2 mAb, are widely used to treat metastatic colorectal cancer (mCRC) and squamous cell carcinoma of the head and neck (SCCHN). While both are effective, they are associated with high rates of skin toxicities, particularly acneiform rash, occurring in almost 90% of patients with 10–20% of patients experiencing grade 3–4 skin toxicity [19]. Other associated grade 3–4 toxicities include infusion reactions (panitumumab < 0.5% vs. cetuximab 2%) and hypomagnesaemia (panitumumab 7% vs. cetuximab 3%) [20]. Notably, in this study, no high-grade cutaneous side effects occurred. One patient experienced a grade 1 potentially drug-related rash that required symptomatic management and did not impact patient treatment. These findings align with the phase 1 study of mAb806, where no dose-limiting toxicities were observed, and the recommended phase 2 dose was established based on a pre-specified dosing limit [13]. However, the small sample size limits the ability to identify uncommon or infrequent toxicities.

Numerous biomarkers have been identified that predict response to EGFR therapies. In non-small cell lung cancer (NSCLC), somatic mutations within the EGFR kinase domain, specifically exon 19 deletions and the L858R point mutation, are the most reliable predictors of sensitivity to tyrosine kinase inhibitors (TKIs). Similarly, in metastatic colorectal cancer, mutations in KRAS or NRAS and BRAF predict lack of response to anti-EGFR antibodies, while high expression of ligands like epiregulin can indicate increased sensitivity [21,22,23,24]. ABT-806 is only able to find its epitope under conditions where EGFR is overactive through mechanisms such as the presence of a range of EGFR oncogenic mutations including EGFRvIII. Because mAb806 and its derivative ABT-806 bind to a cryptic epitope exposed only during EGFR abnormalities, such as overexpression or oncogenic mutations like EGFRvIII and various missense mutations (G574V, R84K, A265V, and G39R), this unique binding profile may serve to enrich for patients likely to respond to ABT-806 treatment or mutation [24,25,26,27]. Consequently, EGFR activation status and associated molecular alterations may represent potential predictive biomarkers of clinical benefit (see Table 2). However, patients in this study were enrolled according to histologic subtype rather than EGFR phenotype. As such, the study was not designed to evaluate ABT-806 in a biomarker-enriched population and cannot adequately determine whether clinical activity may be greater in patients selected for target expression. While no clear association between mAb806 expression and clinical outcome was observed, one patient who experienced prolonged disease stabilization demonstrated strong (3+) mAb806 staining. Definitive conclusion cannot be drawn given our small sample size, intratumoral heterogeneity, and limitations of archival tissue [28]. Further studies with an expanded number of more homogenous samples may be useful and potentially relevant across EGFR-targeting antibodies. With one exception, this study did not identify any EGFRvIII expression in samples tested, which is not unexpected. There is significant variability in the literature on the incidence of the EGFRvIII deletion mutant in HNSCC stemming from the different detection techniques used, with the frequency of EGFRvIII expression ranging from 0 to 75% across studies [29]. Furthermore, EGFRvIII IHC interpretation presents challenges with non-specific cytoplasmic reactivity, lack of standardized scoring systems, and observer variability [29].

ABT-806 and its derivatives have been incorporated into antibody–drug conjugate (ADC) formats using auristatin, pyrrolobenzodiazepines, and Bcl-XL inhibitor payloads. ADCs such as depatuxizumab mafodotin [30], losatuxizumab vedotin [31], serclutamab talirine [32], and ABBV-637 [33] have progressed to clinical trials in both glioblastoma [34] and also other solid tumors [31]. The ability of ABT-806 to target tumors expressing EGFR has been demonstrated using imaging studies, indicating the deliverable concentration to tumor facilitates payload delivery [28]. Of interest, given highly specific targeting of ABT-806, on-going preclinical development of such 806-type drugs in EGFR and other ErbB family would ideally incorporate other payloads such as topoisomerase-1 inhibitors and also further explore a biomarker selection strategy when translated into early clinical trials in the future. Separately, its use as biomarker for ABT-806-directed therapies and its ability to identify the underlying activation status of EGFR also makes it of potential interest in selection of other EGFR-based therapies.

The limitation of our study is the small heterogeneous sample size that precludes any firm conclusion about efficacy and potential equivalence to conventional anti-EGFR therapies in unselected heavily pre-treated patients with squamous cell carcinoma. In addition, our EGFR profiling results utilized archival tumor tissue rather than tissue at the time of study entry. Significant variability in EGFR expression was noted between fresh and archival tissues (FFPE) due to tumor heterogeneity, pre-analytical handling, and biological signaling changes [32]. While archival tissue is the standard for IHC, it often masks the active, dynamic EGFR state that is better captured in fresh, rapid-frozen tissues [32]. We have however demonstrated that ABT-806 is a unique anti-EGFR antibody that was tolerable and, in particular, had negligible rates of traditional anti-EGFR toxicities like rash.

## 5. Conclusions

In a small and heterogeneous population of heavily pre-treated patients, ABT-806 demonstrated a favorable safety profile, albeit with limited efficacy, as monotherapy. The presence of the mAb806-reactive EGFR epitope in tumor specimens supports the utility of mAb806 as a tumor-selective targeting platform. These results provide a rationale for the further development of mAb806-based ADCs and other targeted therapeutic approaches in EGFR-expressing SCC.

## Figures and Tables

**Table 1:** Demographics.

Baseline Characteristics	N = 12 (%)
Age: Median age (range)—year	75.0 (range 62–85)
Sex: Male	9 (75)
ECOG Performance status	
0–1	12 (100)
Tumor type	
Head and Neck (Cohort A)	7
SCC of other sites (cohort B)	5
SCC of other sites (cohort B)	
Lung	2
Skin	1
Anal	1
Renal	1
Prior lines of therapy	
0	1
1	3
2	3
≥3	5

**Table 2:** Mab806 and EGFRvIII expression using immunohistochemistry.

			EGFRvIII				Mab806				Best Response
Cohort (Patient)	Malignancy	Tissue Tested	No Staining	1+	2+	3+	No Staining	1+	2+	3+	
A											
#4	Left pre-auricular SCC	L3 epidural soft tissue	+							+	Stable disease
#5	Parotid gland SCC	Infratemporal fossa	+					+			Progressive disease
#6	Esophageal SCC	Esophagus	+						+		Progressive disease
#7	R-tonsil	Skin (neck)	+				+				Progressive disease
**B**											
#1	Anal SCC	Liver			+				+		Progressive disease
#3	Left forearm SCC	Left Forearm	+				+				Progressive disease
#4	NSCLC	R-pleural tissue	+				+				Stable disease

NSCLC: Non-small cell lung cancer; SCC: squamous cell carcinoma.

## Data Availability

The data generated in this study are available upon request from the corresponding author.

## Supplementary Materials

The following supporting information can be downloaded at: https://www.mdpi.com/article/10.3390/cancers18203313/s1, Table S1: Disease Characteristics of Treated Patients. Table S2: Treatment Emergent Adverse Events.
