# Supplementary material for: An Exploratory Study of ABT-806, a Tumor-Specific Anti-EGFR Antibody, in Subjects with Recurrent or Metastatic Head and Neck and Other Squamous Cell Carcinomas
Source: Cancers (Basel). 2026 Oct 14;18(20):3313. doi: 10.3390/cancers18203313 (PMC13702275; doi:10.3390/cancers18203313)
Supplement: Supplementary file 1 [file cancers-18-03313-s001.zip › Supplementary Table S1.pdf]

Supplementary Table S1: Disease Characteristics of Treated Patients

Patient	Primary Tumor	Disease status at study entry	Sites of metastatic disease
**Cohort A**			
1 (016)	Post-Crocoid SCC	Metastatic	Lung
2 (015)	Tongue SCC	Local (recurrent)	
3 (013)	Tongue SCC	Metastatic	Lymph nodes, Liver
4 (012)	R pre-auricular SCC	Metastatic	Lymph nodes, Lung
5 (010)	Parotid/Skin SCC	Metastatic	Lung
6 (009)	Esophagus	Metastatic	Lymph nodes, Pleural effusion
7 (005)	R-Tonsil	Metastatic	Lymph nodes, pulmonary nodules
**Cohort B**			
1 (011)	Anal SCC	Metastatic	Lymph nodes, Liver
2 (007)	Renal SCC	Metastatic	Lung, Liver
3 (003)	L forearm SCC	Metastatic	Lung, Liver
4 (002)	NSCLC	Metastatic	Lymph nodes, Bone
5 (004)	Lung SCC	Metastatic	Lymph nodes, Liver
