# Supplementary material for: An Exploratory Study of ABT-806, a Tumor-Specific Anti-EGFR Antibody, in Subjects with Recurrent or Metastatic Head and Neck and Other Squamous Cell Carcinomas
Source: Cancers (Basel). 2026 Oct 14;18(20):3313. doi: 10.3390/cancers18203313 (PMC13702275; doi:10.3390/cancers18203313)
Supplement: Supplementary file 1 [file cancers-18-03313-s001.zip › Supplementary Table S2 revised TRACKED.pdf]

Supplementary Table S2: Treatment Emergent Adverse Events

Characteristic CTCAE Term n (%)	Grade 1 N = 23	Grade 2 N = 6	Grade 3 N = 4	Grade 4 N = 0	Relationship to ABT-806
Constipation	2 (9)	2 (32)	0 (0)	0 (0)	Unrelated
Anorexia	2 (9)	0 (0)	0 (0)	0 (0)	Unrelated
Fatigue	5 (22)	0 (0)	0 (0)	0 (0)	Possibly related
Pulmonary Embolism	0 (0)	0 (0)	1 (25)	0 (0)	Unrelated
Scapular Pain	1 (4)	0 (0)	0 (0)	0 (0)	Unrelated
Dysphagia	0 (0)	1 (17)	2 (50)	0 (0)	Unrelated
Cough	0 (0)	1 (17)	0 (0)	0 (0)	Unrelated
Chills	1 (4)	0 (0)	0 (0)	0 (0)	Related
Neutrophilia	1 (4)	0 (0)	0 (0)	0 (0)	Unrelated
Hyponatraemia	0 (0)	0 (0)	1 (25)		Unrelated
Reflux	1 (4)	0 (0)	0 (0)	0 (0)	Unrelated
Thrush	1 (4)	0 (0)	0 (0)	0 (0)	Unrelated
Left Cheek wound Infection	1 (4)	0 (0)	0 (0)	0 (0)	Unrelated
Pain	1 (4)	0 (0)	0 (0)	0 (0)	Unrelated
Headache	1 (4)	0 (0)	0 (0)	0 (0)	Unrelated
Acneiform Rash	1 (4)	0 (0)	0 (0)	0 (0)	Related
Back pain	1 (4)	0 (0)	0 (0)	0 (0)	Unrelated
Gout	1 (4)	0 (0)	0 (0)	0 (0)	Unrelated
Arthritis	1 (4)	0 (0)	0 (0)	0 (0)	Unrelated
Dyspnoea	0 (0)	1 (17)	0 (0)	0 (0)	Unrelated
Infusion Reaction	0 (0)	1 (17)	0 (0)	0 (0)	Related
Diarrhoea	1 (4)	0 (0)	0 (0)	0 (0)	Possibly related
Hypomagnesaemia	1 (4)	0 (0)	0 (0)	0 (0)	Possibly related

Note: Percentages are calculated using the total number of adverse events within each toxicity grade category rather than the number of treated patients. Individual patients may have experienced more than one adverse event.
